# RETRACTION: Impact of Stapler Versus Manual Closure Techniques on Pharyngocutaneous Fistula Development Post‐Total Laryngectomy: A Systematic Review and Meta‐Analysis

**DOI:** 10.1111/iwj.70618

**Published:** 2025-04-21

## Abstract

**RETRACTION:** DingS. , ZhangY. , GuoW. , YinG. , HuangZ. , and ZhongQ. , “Impact of Stapler Versus Manual Closure Techniques on Pharyngocutaneous Fistula Development Post‐Total Laryngectomy: A Systematic Review and Meta‐Analysis,” International Wound Journal 21, no. 3 (2024): e14751, 10.1111/iwj.14751.38472132 PMC10932772

The above article, published online on 12 March 2024, in Wiley Online Library (http://onlinelibrary.wiley.com/), has been retracted by agreement between the journal Editor in Chief, Professor Keith Harding; and John Wiley & Sons Ltd. Following an investigation by the publisher, all parties have concluded that this article was accepted solely on the basis of a compromised peer review process. The editors have therefore decided to retract the article. The authors disagree with the retraction.

**RETRACTION:** S. Ding , Y. Zhang , W. Guo , G. Yin , Z. Huang , and Q. Zhong , “Impact of Stapler Versus Manual Closure Techniques on Pharyngocutaneous Fistula Development Post‐Total Laryngectomy: A Systematic Review and Meta‐Analysis,” International Wound Journal 21, no. 3 (2024): e14751, 10.1111/iwj.14751.38472132 PMC10932772

The above article, published online on 12 March 2024, in Wiley Online Library (http://onlinelibrary.wiley.com/), has been retracted by agreement between the journal Editor in Chief, Professor Keith Harding; and John Wiley & Sons Ltd. Following an investigation by the publisher, all parties have concluded that this article was accepted solely on the basis of a compromised peer review process. The editors have therefore decided to retract the article. The authors disagree with the retraction.

